# Study flow diagrams in Cochrane systematic review updates: an adapted PRISMA flow diagram

**DOI:** 10.1186/2046-4053-3-54

**Published:** 2014-05-29

**Authors:** Elizabeth Stovold, Deirdre Beecher, Ruth Foxlee, Anna Noel-Storr

**Affiliations:** 1 Cochrane Airways Group, St George’s, University of London, Cranmer Terrace, Tooting, London SW19 2HG, UK; 2 Cochrane Injuries Group, London School of Hygiene & Tropical Medicine, Keppel Street, London WC1E 7HT, UK; 3 Cochrane Editorial Unit, 11-12 Cavendish Square, London W1G 0AN, UK; 4 Cochrane Dementia and Cognitive Improvement Group, Radcliffe Department of Medicine, University of Oxford, John Radcliffe Hospital, Headington, Oxford OX3 9DU, UK

**Keywords:** systematic reviews, review updates, literature searching, study flow diagrams, reporting standards, PRISMA statement

## Abstract

Cochrane systematic reviews are conducted and reported according to rigorous standards. A study flow diagram must be included in a new review, and there is clear guidance from the PRISMA statement on how to do this. However, for a review update, there is currently no guidance on how study flow diagrams should be presented. To address this, a working group was formed to find a solution and produce guidance on how to use these diagrams in review updates.

A number of different options were devised for how these flow diagrams could be used in review updates, and also in cases where multiple searches for a review or review update have been conducted. These options were circulated to the Cochrane information specialist community for consultation and feedback. Following the consultation period, the working group refined the guidance and made the recommendation that for review updates an adapted PRISMA flow diagram should be used, which includes an additional box with the number of previously included studies feeding into the total. Where multiple searches have been conducted, the results should be added together and treated as one set of results.

There is no existing guidance for using study flow diagrams in review updates. Our adapted diagram is a simple and pragmatic solution for showing the flow of studies in review updates.

## Background

A Cochrane systematic review is conducted according to rigorous methods outlined in the Cochrane Handbook [1]. The review begins with a protocol to define the question, the inclusion and exclusion criteria, and the proposed methods, including a comprehensive search strategy to identify relevant studies. Cochrane protocols and the completed reviews are published in the Cochrane Database of Systematic Reviews on *The Cochrane Library*[2]. The Methodological Expectations of Cochrane Intervention Reviews (MECIR) conduct and reporting standards [3], published in 2012, explicitly set out the criteria a Cochrane review must meet if it is to be published on *The Cochrane Library*. Included in these standards is the requirement that all new Cochrane reviews must include a PRISMA study flow diagram [4].

A distinguishing feature of a Cochrane review is that it is updated regularly in an effort to ensure that the most recent evidence is incorporated [5]. Historically, the aim was to update Cochrane reviews every two years [6], but recently there has been a move away from this policy in favour of prioritising the most clinically important reviews for updating [7]. In some instances a review no longer requires updating, for example if the treatment is no longer current, in which case it is deemed to be a ‘stable’ review. In other cases, new trial evidence continues to emerge over many years, and reviews must be updated multiple times. At present, the MECIR standards only require new reviews to include a PRISMA diagram, but in practice, many author teams do try to incorporate these diagrams into review updates. The original review may already contain a PRSIMA diagram, but generally this is not the case because the majority of existing reviews were produced before the MECIR standards were published.

For a new review, use of the PRISMA diagram template as shown in the PRISMA statement [4] is recommended. For a review update, the situation is more complex as the diagram needs to take into account studies included when the review was first published, plus any new studies identified in the update. To our knowledge, there is not any published guidance on how to show this in a PRISMA diagram. This led us to develop an adapted diagram specifically for use in a review update.

## Main text

### Aims

The aim of this publication is to create guidance for using a PRISMA diagram in a review update that clearly shows the search activities performed during the entire lifecycle of a review and the decisions made on the inclusion and exclusion of studies for that update.

### Process

At the 2012 annual meeting of the United Kingdom-based Cochrane information specialists, there was a discussion about how to incorporate study flow diagrams into review updates, which was prompted by an increasing number of queries from review authors who were seeking guidance on this issue. Following the meeting, the authors of this paper, all information specialists who work with different Cochrane review groups, formed a working group to consider this topic.

There are a number of different situations that arise when a review is updated in relation to the included studies. The original review may already have included studies, but this is not always the case. The review update may identify new studies for inclusion, but equally, it may not. Our study flow diagram had to be able to cope with all of these situations. Multiple searches are often conducted before a review or review update is finalised, so we needed to consider how that might be shown in the diagram as well. With this in mind, we developed a series of flow diagrams, setting out the advantages and disadvantages for each model, and circulated these to the wider Cochrane information specialist community for consultation. The issue was raised by review authors looking to Cochrane information specialists for advice, and for that reason, we decided to consult with this group.

**Figure 1:**
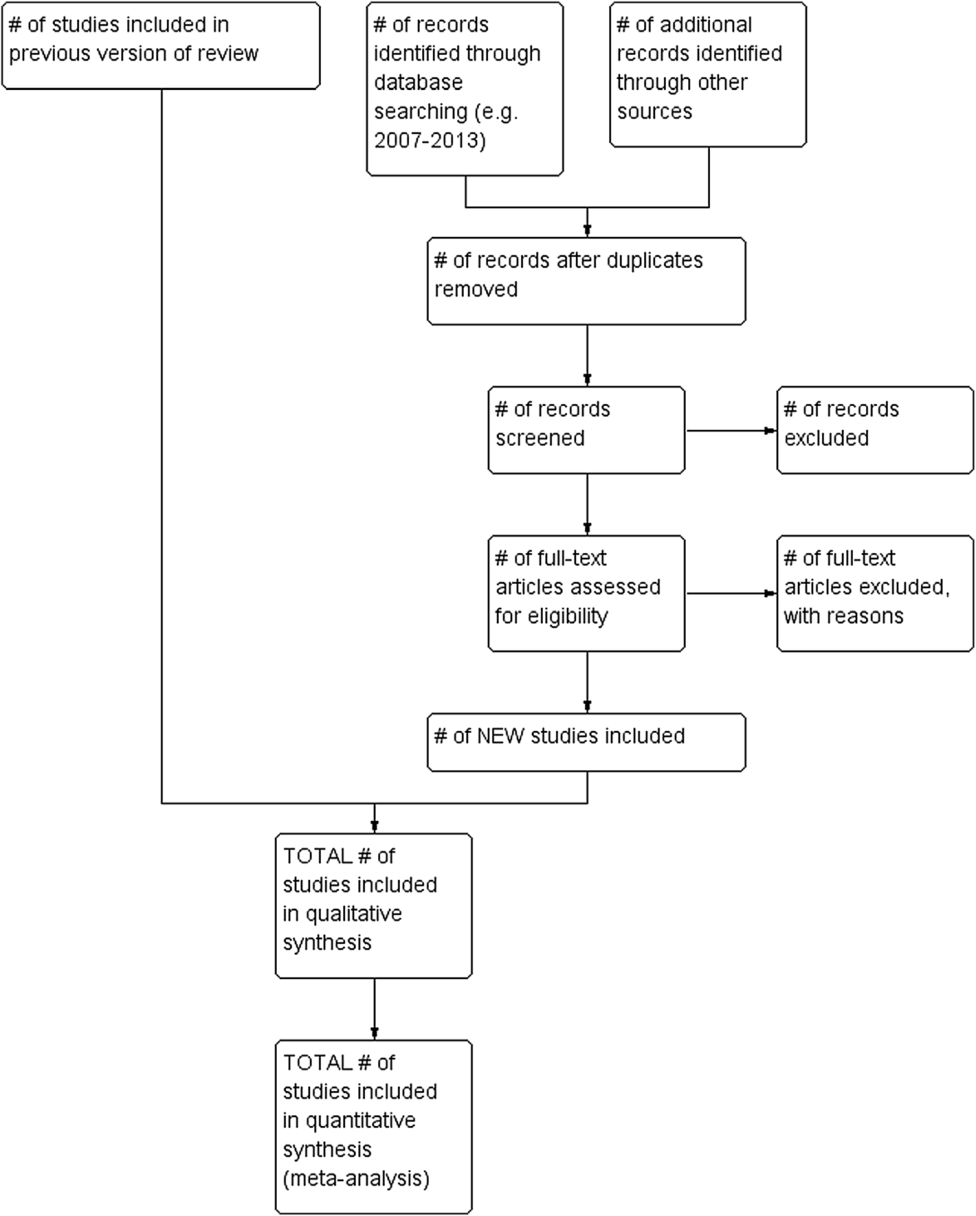
**Adapted study flow diagram.** Study flow diagram for a review update with previous included studies incorporated into the results of an updated literature search.

For review updates, the following three models for a study flow diagram were presented and refined during the consultation period:

1. A separate PRISMA diagram for each review update, showing the search results, screening and inclusions for that update only. Generating a separate diagram for each review update is the easiest option; however, this would result in multiple diagrams as subsequent updates are published, and would not show the total number of included studies very clearly.

2. A cumulative diagram, where all search results from the original review and subsequent updates are added together in a single figure. As in model 1, this diagram would be simple to generate, but it would not be possible to identify the number of new studies that were included in each individual update.3. A single diagram for the current review update, with the number of included studies from the original review or previous update included in the total. By adding an extra box at the top of the PRISMA diagram template (Figure 1) the number of included studies from the original review or previous update, and the new studies identified for the current update can be clearly shown. However this model does not show the total number of references screened for the review overall.

Option 3 was selected by the working group as our preferred option as this model best reflected the study identification process for the current review update. This option is particularly useful when the search history of the original review or previous update is incomplete and does not already contain a study flow diagram, which is the case for many reviews due for updating at the time of writing.

The options for dealing with multiple searches for both new reviews, and review updates were similarly laid out:

1. A single diagram where results from multiple searches would be added together**.** This is the simplest option, summarising the searching activity for the reader in the least complicated way. It is not a true reflection of the searching activity, in that multiple searches will almost certainly have been conducted; however, the dates of individual searches could be included in the diagram if desired.

2. A single diagram, but with the results of individual searches (for example, the initial search, search update and pre-publication search) represented in the diagram. This format would clearly show the results of multiple searches and it would be easy to add extra boxes for each search, but it would complicate the diagram with the potential for a large number of extra boxes.

3. A separate diagram for each search. This format will show the full screening and exclusion process for each search update; however, this could result in a large number of figures being generated, and it would not necessarily be clear how many studies are included in total.

Option 1 was selected as our preferred option because it shows the searching activity in the simplest and clearest way.

## Results

During the feedback period of four weeks, we received a response from 7/52 (13%) of the Cochrane information specialists. Overall, six of the seven responders agreed with our preferred options and the discussion document was subsequently revised to summarize our recommendations. While the seventh respondent agreed with the use of our adapted diagram when the search history of the original review or previous update is unclear, this respondent suggested that a review could contain both a cumulative diagram showing the overall search history of the review and a diagram for the current update. This would involve the publication of two diagrams and each subsequent update would require the addition of a new diagram, which could become unwieldy. Nevertheless, this is an idea that some Cochrane groups or individual review teams may wish to consider.

Following the consultation period, the working group recommended that:

1. Where multiple searches have been conducted for a review or review update, the results of all searches should be added together.2. For a review update, two extra boxes will be added: one for the number of studies included in the original review or previous update and one for the new studies retrieved for the current update (Figure 1). If multiple searches have been conducted for the current update, the results of all the searches should be added together.

These recommendations were forwarded to the Cochrane Methods Coordinator and will inform the revision of the ‘Review Update’ chapter of the Cochrane Handbook. We are encouraging review authors working with our respective Review Groups to try our adapted diagram in their review updates, and we intend to disseminate this work to our colleagues in other Cochrane Groups.

## Discussion

At present, the Cochrane standards only require new reviews to include PRISMA diagrams; however, taking a best practice approach, it is clear that this should apply to review updates as well. This paper has outlined the process undertaken by Cochrane information specialists to develop guidance for using a PRISMA diagram that can be applied to review updates, whether there are previously included studies or not, together with any new included studies.

This work has been a first step, and there are still issues to consider. The adapted diagram does not clearly show the total number of references that have been screened over the lifetime of the review. In fact, this information may not be available, as the majority of reviews were first written before the current standards for reporting search histories came into force. However, if our recommendations are followed, the number of included studies in the previous version would be clearly shown, together with the number of newly identified included studies. Each subsequent update will contain an updated version of this diagram. The original review and any previous updates are archived on *The Cochrane Library*, so it will always be possible to refer back to the previous diagrams if needed.

Whilst the main driver for this project was to identify a solution for documenting the flow of studies in review updates, the issue of multiple searches conducted for a review or review update proved to be a complicating factor. We determined that the simplest and clearest way to deal with this is to sum the results of all the individual searches from one review version together and treat them as one set of search results, whilst acknowledging that this will not always accurately reflect the searching activity that has taken place.

This work was conducted in response to queries from review authors working with Cochrane Review Groups. The methods we used to produce our adapted diagram were pragmatic in order to solve a problem arising in our day-to-day work. We did not attempt to follow a formal method for the development of reporting guidance as this was beyond the scope of the time and resources available to us, and we did not consult widely outside the Cochrane information specialist community. We received a low response rate from the consultation, which is a limitation of this paper; however, we hope that further work will be carried out on this important issue. An evaluation of the uptake and use of this adapted diagram, a survey of other methods used, and wider consultation with systematic review authors, editors and other interested parties would all be valuable projects.

## Conclusion

There is a lack of guidance on how to report the flow of studies in PRISMA diagrams for review updates. Our adapted PRISMA diagram is a simple and pragmatic solution for showing the flow of studies through a Cochrane or non-Cochrane review update. Further work should be conducted in this area to evaluate the use of this diagram and to seek feedback from a wider audience.

## Abbreviations

MECIR: Methodological Expectations of Cochrane Intervention Reviews conduct and reporting standards; PRISMA: Preferred Reporting Items for Systematic Reviews and Meta-Analyses.

## Competing interests

ES, DB and ANS are currently employed by a Cochrane Review Group as information specialists. RF was previously employed by a Cochrane Review Group as an information specialist and is now employed by the Cochrane Editorial Unit, also as an information specialist.

## Authors’ contributions

ES convened the working group, contributed to the consultation document, collated feedback, wrote the draft manuscript, and critically revised and approved the final manuscript. DB participated in the working group, contributed to the consultation document, and critically revised and approved the final manuscript. RF participated in the working group, contributed to the consultation document, and critically revised and approved the final manuscript. ANS participated in the working group, contributed to the consultation document, and critically revised and approved the final manuscript. All authors read and approved the final manuscript.
